# Visual search strategies and game knowledge in junior Australian rules football players: testing potential in talent identification and development

**DOI:** 10.3389/fpsyg.2024.1356160

**Published:** 2024-06-06

**Authors:** Lael Kassem, Bonnie Pang, Sera Dogramaci, Clare MacMahon, John Quinn, Kylie A. Steel

**Affiliations:** ^1^School of Health Sciences, Western Sydney University, Sydney, NSW, Australia; ^2^Department for Health, University of Bath, Bath, United Kingdom; ^3^Sport, Performance, and Nutrition Research Group, Latrobe University, Melbourne, VIC, Australia; ^4^Greater Western Sydney Giants AFL, Sydney, NSW, Australia; ^5^Quinn Elite Sport Services, Sydney, NSW, Australia; ^6^The MARCS Institute for Brain, Behaviour and Development, Sydney, NSW, Australia

**Keywords:** talent identification and development, Australian rules football, eye-movement behavior, decision-making, sport expertise

## Abstract

This study explored video-based decision-making and eye-movement behavior as a complementary method to assess the decision-making skills and knowledge of elite junior Australian Rules (AR) Football players. Performance was measured twice over an 18-month period. This approach tested a practical and reliable assessment of decision-making and game knowledge that does not contribute to physical training load. *N* = 59 participants were categorized based on their training age groups, U14 (*N* = 38, *M_age_*13.37 ± 0.47) and U16 (*N* = 21, *M_age_*14.80 ± 0.39). Participants watched 14 brief video clips and provided action choices while wearing eye-movement recording glasses that captured visual search patterns (e.g., fixations). Decision accuracy and speed of decision-making were also recorded. Participants with accurate decisions made significantly faster decisions compared to less skilled players (*p* < 0.001). Further, skilled participants had significantly fewer fixations of shorter duration compared to less skilled participants at both the initial and follow-up testing sessions (*p* < 0.0001). This suggests that eye-movement characteristics, remain a relatively stable measure over moderate periods of time. With the ability to differentiate between more and less skilled decision-makers, this proof-of-concept study proposes that examining eye movements in relation to decision-making and game knowledge is a viable tool for Talent Identification and Development (TID) to complement current measures. We provide a platform for further development and research in the quest for efficient and effective talent identification processes.

## Introduction

The ability to identify performers with a high potential for development within junior team sports is challenging as it requires the consideration of a range of multidimensional elements (Falk et al., 2004; Farrow and Raab, 2008). These elements include physiological, physical, psychological, technical, and tactical variables (Light and Harvey, 2019). Another challenge in talent identification and development (TID) programs is forecasting elite performance over the course of a junior athlete’s development into adult competition, given the influence of variable rates of maturation, individual learning and coaching styles, and the volume of and opportunity for practice. These challenges drive continued efforts to evolve TID systems to ensure that a club’s resources are invested for the most suitable athletes.

The Australian Football League (AFL), which operates the Australian Rules (AR) football competition, uses a cross-sectional design to identify talented junior athletes with potential. A cross sectional approach assumes that identified sport-specific characteristics develop in a “linear” manner from elite junior to elite senior contexts (Güllich and Emrich, 2012) and tests athletes within the same age groups across a range of sport-specific performance measures. Talent-identified junior athletes are then invited to participate in development programs provided by regional and state-based academies. During these development programs, athletes are exposed to expert coaching and specific interventions designed to accelerate the acquisition of sport-specific skills required for success in elite senior competitions (Matthys, 2012; Johnston et al., 2018). The talent development program also exposes athletes to a greater volume of effortful, work-like practice and structured training, which some research shows facilitate elite performance (Ericsson et al., 1993; Ford et al., 2009). However, state-based academies can only take in a limited number of athletes who demonstrate the potential to achieve elite levels of performance. Hence with limited places in these programs, it is critical that the TID process is effective and efficient in its selections while also minimizing talent wastage.

Despite the necessity for efficiency in TID procedures, some limitations remain. First, athlete testing remains focused on the physical and physiological attributes of the individuals (e.g., speed and technical ability) (Burgess et al., 2012; Woods et al., 2016). Current practice also does not sufficiently consider the influence of variation in maturation status, and that chronological age and biological maturity do not develop at the same rate for all individuals. For example, a 15-year-old athlete may have a biological maturation above or below their actual age, which could influence performance characteristics (Beunen et al., 2000; Cripps et al., 2017; Gogos et al., 2020). Moreover, the relative age effect, in which birthdate relative to selection year for sport influences progression (Kelly et al., 2024), may amplify or moderate variations in maturation status. In addition, given the dynamic nature of sports, athletes tend to compensate for different strengths and weaknesses. For example, skilled decision-makers may use this ability to compensate for any physical and/or technical skill deficiencies (Wolstencroft and House, 2002; Woods et al., 2016).

A further limitation of current TID practices in AR football relates to how perceptual cognitive skills such as decision-making are assessed. Specifically, making fast and accurate decisions is a critical skill in AR football (Farrow et al., 2008; Woods et al., 2016), and requires a player to accurately select the correct choice from a range of alternatives under varied environmental contexts (Bruce et al., 2012; Dicks et al., 2019). Effective sport decision making also requires the integration of perceptual, cognitive, and motor skills (Gréhaigne et al., 2001), and in team sports, the ability to learn the team’s particular game play and decision-making style as directed by the coach. This latter declarative knowledge is influential at junior stages of development and sport pedagogy (Dong et al., 2023).

Assessment of the tactical decision-making and declarative knowledge component of gameplay remains subjective. Indeed, the primary means of assessing decision-making is for recruiters to observe the performance of players in gameplay and pass judgment (Burgess et al., 2012; Larkin et al., 2020). While a primarily subjective process has its place (MacMahon et al., 2019), more objective assessments of perpetual-cognitive skills such as decision-making can complement subjective assessments and increase efficiency in TID efforts. In addition, junior athletes continue to develop declarative game knowledge, as an essential foundation for effective skill and tactical learning within professional contexts, and for general perceptual-cognitive skill (Américo et al., 2017). It is the challenge of assessing junior athletes’ game knowledge and decision making through a more objective measure which this research aims to address.

To help understand the assessment of decision making and game knowledge, it is useful to dissect the underpinning processes. Decision-making is influenced by the ability to perceive and integrate complex moving patterns while allocating attentional resources to different key areas of dynamic plays, to make appropriate action choices (Williams and Jackson, 2019). Research has shown that three main factors underpin perceptual-cognitive skill, including visual search strategy, processing cognitive information, and anticipatory expectations within the display (Farrow et al., 2008; Woods et al., 2016). Visual search is most relevant to this study and may be measured relative to decision-making and game knowledge to improve TID programs. For example, research shows that skilled decision-makers use more efficient visual search strategies, and faster cognitive processing is linked to advanced cue utilization (Cardoso et al., 2021). These processes allow the identification of familiar patterns and key areas of importance within a display, enhancing anticipation (Allard and Starkes, 1980; Abernethy, 1990; Güllich and Emrich, 2006; Afonso et al., 2014; Williams and Jackson, 2019; Vater et al., 2020).

While several studies have attempted to quantify decision-making skill within AR football (Lorains et al., 2013; Woods et al., 2016), few have investigated the decision-making skills of junior athletes and the possible influence of maturation stages on this skill.

This study expands on previous findings, by using a film-based decision-making task and eye movement registration to explore the decision-making abilities and processes of elite junior AR football players. Specifically, this study explores the relationship between eye movement behavior and decision-making when differentiating more and less skilled junior decision-makers. Laurent et al. (2006) state skilled athletes exhibit fewer fixations and are more likely to identify and recognize more meaningful patterns in various scenarios more accurately when compared to less skilled players. Therefore, we hypothesized that skilled decision-makers would demonstrate more efficient eye-movement behaviors incorporating fewer fixations on redundant information, and more fixations of shorter duration on relevant cues when compared to less skilled decision-makers (Brams et al., 2019; Vitor de Assis et al., 2020).

## Methods

### Participants

Fifty-nine junior male AR football players were recruited for this study from an elite state football academy. At the time of testing no female squad had been established thus we were restricted to male players only. Players were members of the development squad for the 2018 season. Participants were categorized based on their training age groups, U14 (under 14 years) (*n* = 38, *M_age_*13.37 ± 0.47) and U16 (under 16 years) (*n* = 21, *M_age_*14.80 ± 0.39). Eighteen months later at the follow-up stage, only 18 of the original 59 participants were still members of the academy programs due to dropout or deselection. This second subsample provided an assessment of a relatively more successful subgroup. At the follow-up, participants were categorized in the same manner as the initial testing, that is respective to age U16 (*n* = 13, *M_age_*14.91 ± 0.48) and U18 (*n* = 5, *M_age_*16.40 ± 0.19). All participants provided informed consent before involvement with the study following the ethics protocol approved by the University Human Research Ethics Committee.

#### Video footage

The video footage for testing was provided by the professional AR football club involved in this study and was from stored game-day footage captured during the 2017 and 2018 seasons. Two different viewing perspectives were available: behind the goals and broadcast (televised footage). This ensured that all clips portrayed an offensive play. Players had between three and five options to choose from, with a lead time of 15 s before the decision-making moment (Woods et al., 2016). The initial videos were sourced from the 2017 season, while the follow-up group had a new set of videos sourced from the 2018 season.

#### Decision-making options

A total of 40 videos were identified for use in this study and reviewed by three highly experienced coaches, each with a minimum of 10 years of coaching at state or higher levels. Each coach was asked to identify the best passing options for the player in possession of the ball. Coaches independently ranked their top three options based on a 3–2–1–0 scale. Three represented the most “ideal” option and one represented the “least ideal,” but still acceptable option. Zero points were awarded for any other “unacceptable” option. Only videos where all three coaches agreed upon the three options were included. The same coaches reviewed both initial and follow up video clips. From the range of clips provided to the coaches, 14 videos fitted the criteria to use in the initial testing, while a second set of videos fitting the same criteria were used in the follow up study.

#### Eye-tracking technology

TobiiPro Glasses 2 (Tobii AB, Stockholm, Sweden) eye-tracking glasses were used to measure the eye-movement behavior of the participants during the film-based decision-making task. The glasses collected visual search data at a rate of 50 Hz with 82° horizontal and 52° vertical visual angle of precision. The Tobii Pro Analyzer software program coded all recordings, which produced data for the following variables:

- Decision Time (DT): measured from the moment the video paused at the critical decision-making moment until the participant made a verbal response.
- Number of fixation(s): from the critical decision-making moment until participants made the first verbalization indicating their decision.
- Duration of fixation(s): Each coded fixation automatically also recorded the duration of fixation (across all locations).
- Total duration: the sum of all individual fixation durations.

In addition, the visual display was allocated into one of four areas: Defenders (D), Options (O), Player with the ball (B), and Space (S) for further analysis into participants’ gaze behaviors.

#### Procedures

Testing was conducted across both regional and rural training locations which are within the geographical catchment area for the professional football club involved in this study. On arrival, the chief investigator read a dialogue sheet providing the details of the procedure. With consent, participants were fitted with eye-tracking glasses, followed by individual calibration. Participants stood in front of the screen, ensuring optimal positioning. If required, the height and distance measurements were adapted to each participant, ensuring eyesight was in the center of the display, allowing for the greatest compatibility.

**Figure 1:**
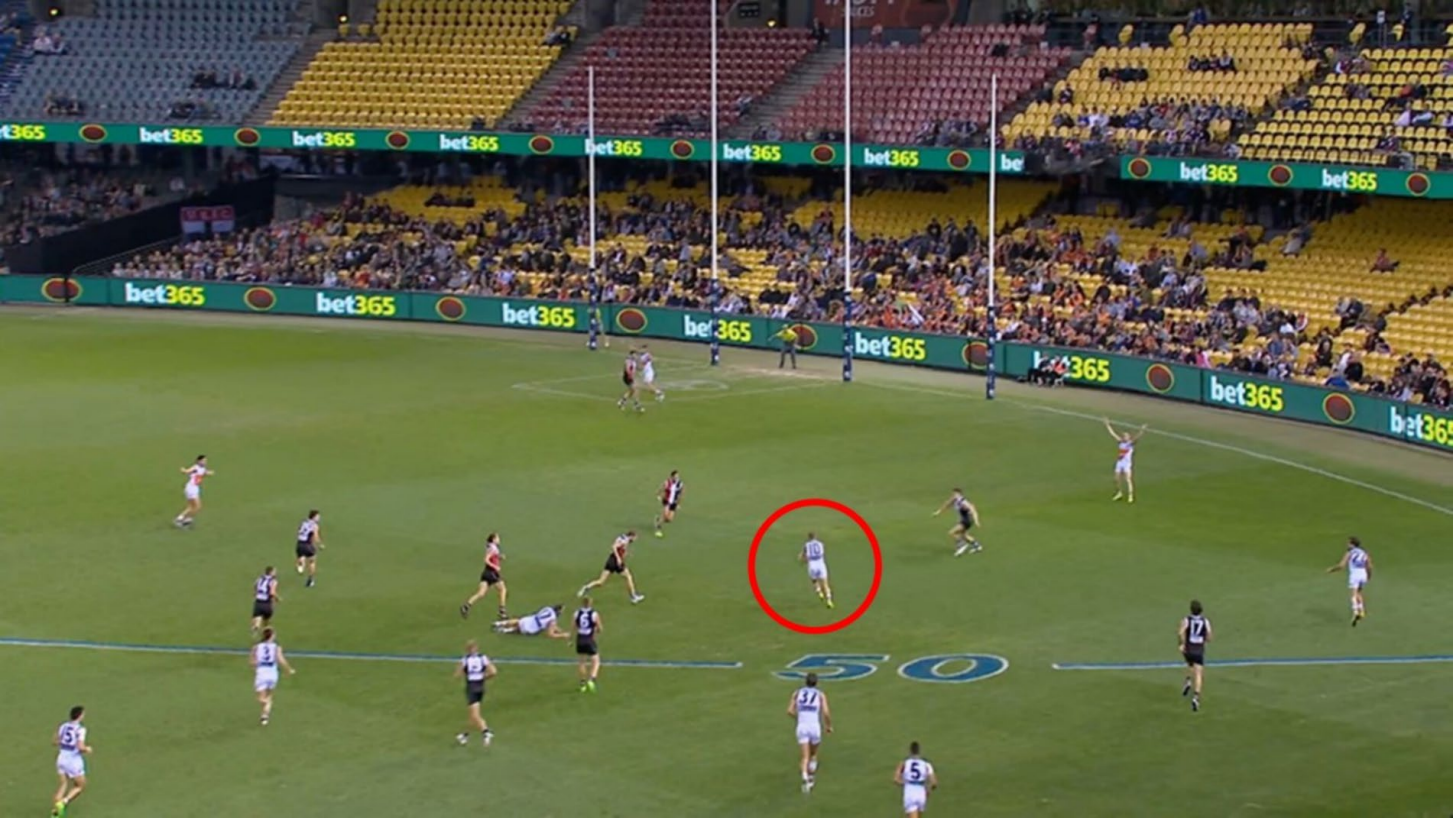
An example of a critical decision-making moment, used for the film-based task. For this example, the player with the ball is circled for reference. The team in dark the colored uniforms is the offensive team.

Before the commencement of the task participants were told they would watch 14 individual clips of game day footage. At a critical point in play, the video clip would stop at which point participants were instructed to state what decision they would make if they were the player in possession of the ball. They were also told that they would only have a four-second window to verbalize (Woods et al., 2016). To ensure participants understood the process they watched two familiarization clips recorded from a similar viewpoint as the test clips. Once ready, participants viewed each of the 14 randomly sequenced clips providing their decision after each clip. The participant’s score was matched with the scale (3–2–1–0) agreed upon by the three independent coaches. Participants received a 0 if they selected an option outside of the coach’s identified appropriate options or if they did not make decisions within the allocated 4 s. An example of the final critical moment of a video clip is shown in Figure 1.

### Statistical analysis

All results were collated, and various statistical procedures were implemented using the Statistical Package for Social Sciences (SPSS, Version 22). Descriptive statistics for all variables were calculated and are reported as mean ± standard deviation (SD). An alpha level of *p <* 0.05 was selected as the criterion for significance for all statistical procedures. A mixed model binomial logistic regression was used to account for the multiple responses, allowing the variance between videos to predict whether players can be correctly classified (skilled v. less skilled) from the independent variables.

Participants were initially divided into age groups of U14 (*n* = 38) or U16 (*n* = 21), based on a biannual age group system used within state academies for the initial testing phase. At the follow-up however, data were grouped as one age cohort, due to the small numbers and close ages (*n* = 13 for U16; *n* = 5 for U18). While this drop in participant numbers created a limitation to the data, the elite nature of the sample which naturally decreases as you reach the elite level of competition warrants continued analysis. Response accuracy (RA) was then used to stratify participants based on their decision-making. We categorized responses into two groups to allow for comparison and to predict the probability of observations falling into the skilled group (3 = Skilled; 2, 1, 0 = Less Skilled). To examine the potential of eye-movement data to predict skill level, a Receiver Operator Characteristic (ROC) curve was used to indicate the players’ level of ability and to predict skilled decision-makers.

Two researchers independently coded the eye movement data via Tobii Pro Analyzer, which involved manually mapping fixation points and allocation of the different areas of interest (AOIs). Once coding was complete, the data was checked for consistency and agreement in coding locations and categories. The level of agreement between coders was very high (>95%). Where differences in coding occurred, the raters discussed and clarified the code.

## Results

### Decision time

#### Initial test session group analysis

**Figure 2:**
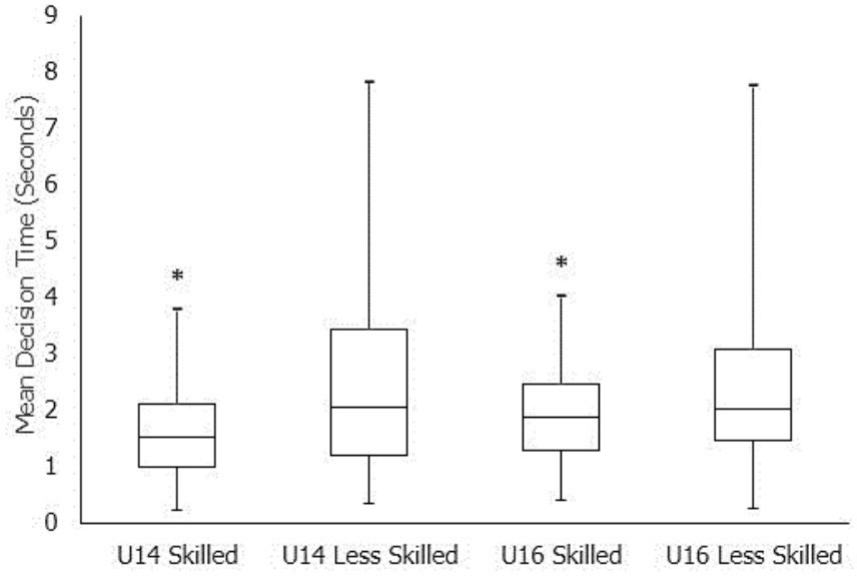
Mean decision time(s) and standard deviation between skilled and less skilled participants across both age groups. *Significantly different compared to the less skilled group (*p* < 0.05). Mid-line represents the median time for that group. Also note that the less skilled groups in each age range exhibited the greatest variation in decision time.

On average, the skilled participants within both U14 and U16 groups made significantly faster decisions (U14: 1.67 ± 0.87 vs. 2.58 ± 1.87; U16: 1.94 ± 0.84 vs. 2.46 ± 1.57) (Figure 2). A binomial logistical regression analysis indicated that for every 1 s increase in DT, there is 0.640 reduced likelihood of being in the skilled group (*p* = 0.001, 95%CI [0.3.02, 0.507]). The model also highlighted between age-group differences, with the older group more likely to be faster when making skilled decisions (*p* = 0.023, 95%CI [0.486, 0.947]).

#### Follow-up test session analysis

**Figure 3:**
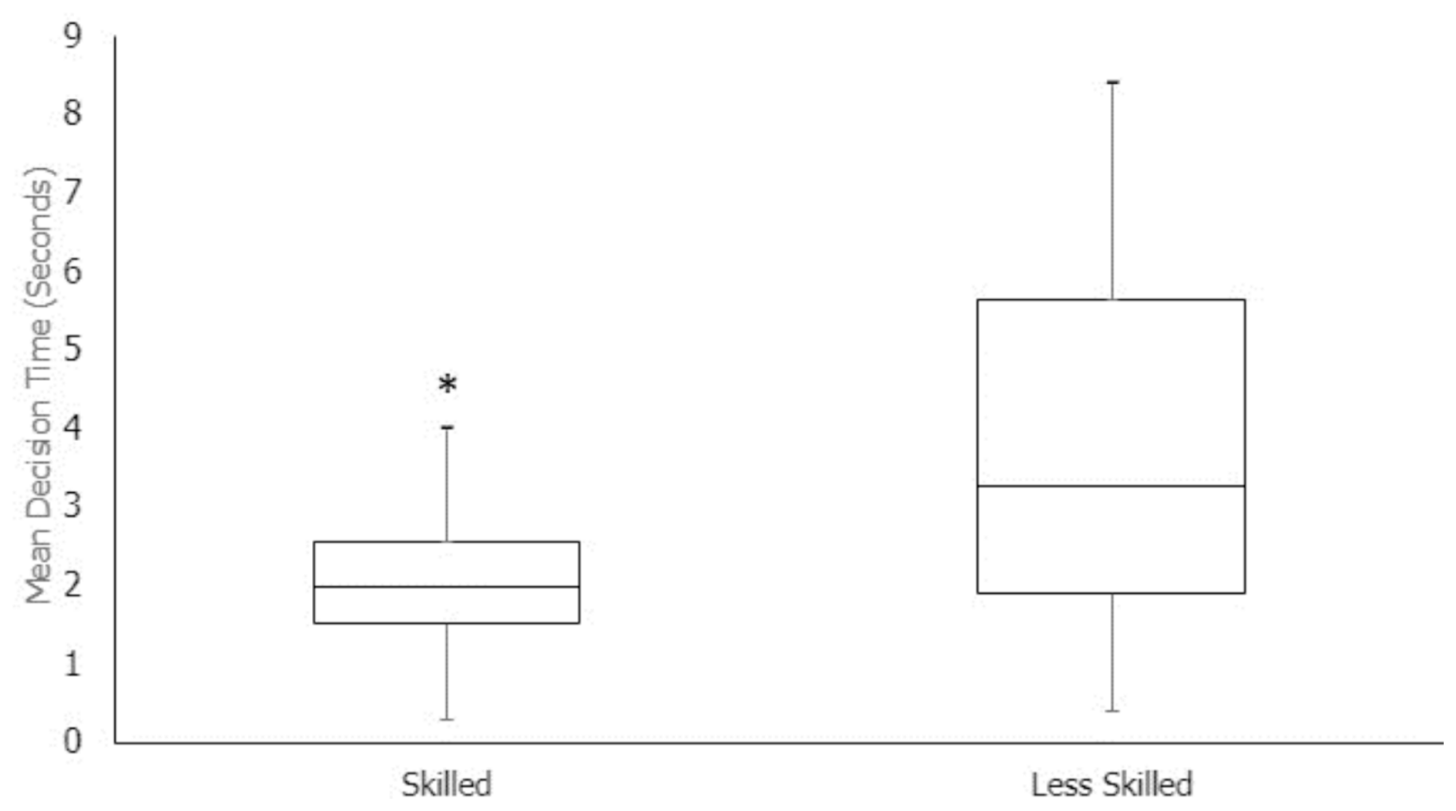
Mean decision time (seconds) between overall skilled and less skilled participants across all follow-up participants. *Significantly different from the less skilled group (*p* < 0.05). Mid-line represents the median time for that group.

At the follow-up testing session, participants demonstrated a similar pattern to that in the initial test, where skilled participants made decisions significantly faster (*p* < 0.05) compared to less skilled participants (2.06 ± 0.80s vs. 3.84 ± 2.16 s, respectively) (Figure 3). The mixed model logistic regression indicated that for every 1-s increase in decision time, the likelihood of being in the skilled group reduced by 0.48[CI 95% 0.370, 0.624]. When compared to the initial test session group, overall, the follow-up test session participants made slower decisions compared to the initial responses (2.84 ± 1.78 s vs. 1.82 ± 1.17 s, respectively). However, this time difference was not significant and did not result in a more skilled response accuracy.

### Eye-movement behavior—initial test session participants

#### Number and duration of fixations

Visual search strategy (number of fixations and total fixation duration) was significantly different between skilled and less skilled groups as shown in Table 1. Specifically, the skilled groups had fewer fixations, and shorter fixation durations than the less skilled groups, in both age groups.

**Table 1:** Mean number and total duration of fixations between skilled and less skilled groups across both age groups.

	Number of fixations (n) Mean ± SD		Fixation duration (s) Mean ± SD	
	U14	U16	U14	U16
Skilled	3.27 ± 1.99*	3.96 ± 2.34*	1.79 ± 1.14*	2.11 ± 1.26*
Less skilled	4.17 ± 2.56	4.46 ± 2.31	2.16 ± 1.37	2.29 ± 1.46

*Significantly different from the less skilled group (*p* < 0.05).

As Table 1 shows, a binomial logistical regression revealed that for every increase in fixation, participants reduced their likelihood of being in the skilled group by 0.863 95%CI [0.804, 0.927] (*p* = 0.0001). Further, the U16 group were more likely to make skilled decisions (*p* = 0.0195%CI [0.467, 0.903]) compared to the U14 group.

Moreover, a binomial logistic regression revealed that for every 1 s increase in duration, the participants reduced the likelihood of being in the skilled by 0.839 95%CI [0.735, 0.956] *p* = 0.008. Analysis also revealed the U16 cohort were more likely to make skilled decisions, *p =* 0.018 95%CI [0.487, 0.934].

#### Fixations across AOIs

**Figure 4:**
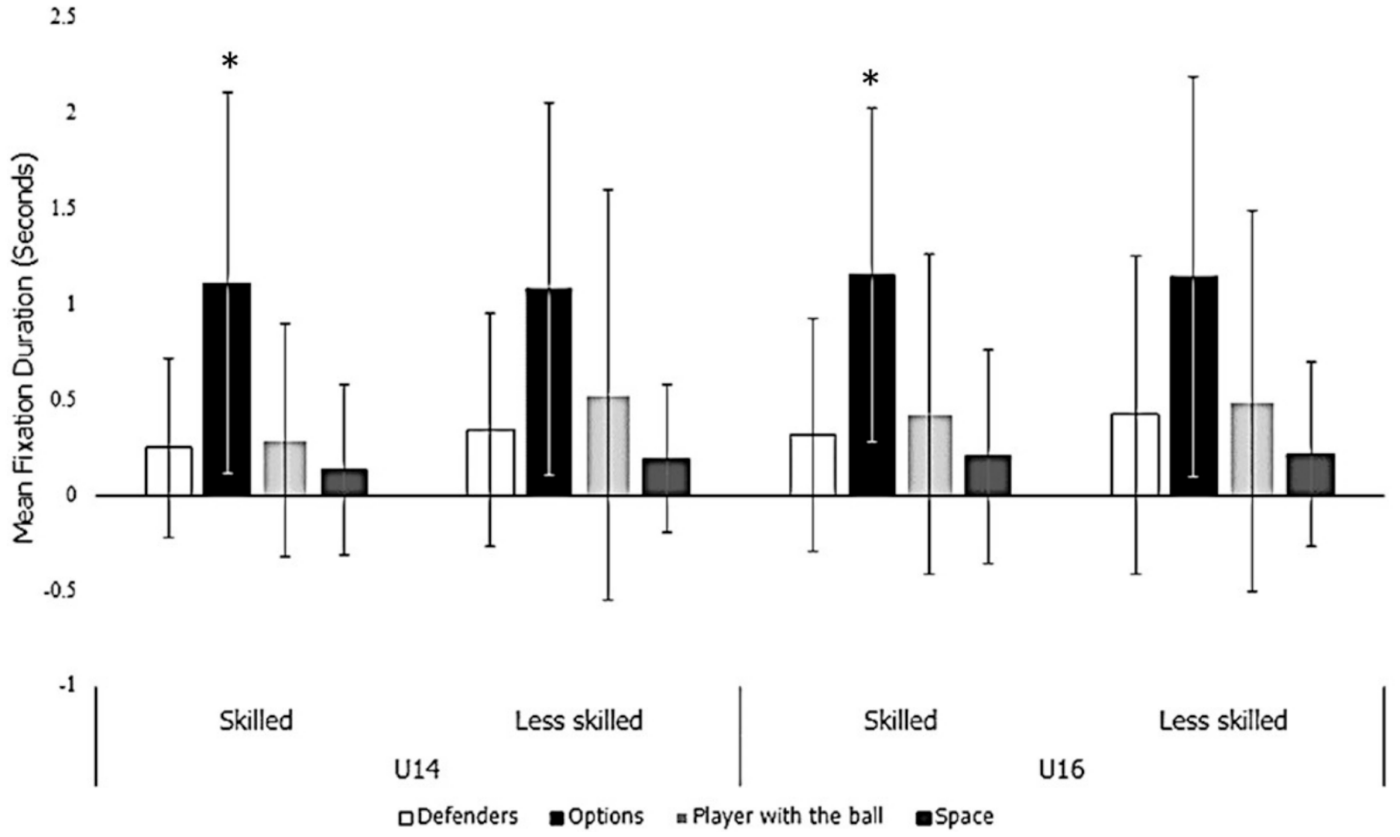
Average fixation duration (seconds) across fixation locations between skilled and age groups. *Significantly different from the less skilled group (*p* < 0.05).

Skilled participants displayed significantly greater fixations on viable options (Figure 4). A binomial logistic regression revealed that each increase in fixation rate/time per decision option correlated with being a member of the skilled group by 0.829 95%CI [0.743, 0.924] (*p* = 0.001).

### Eye-movement behavior—follow-up test session participants

The binomial logistic regression indicated that the number of fixations participants made, and the total duration of fixations were significantly different between initial and follow-up testing (Table 2).

**Table 2:** Mean duration of fixations between skilled and less skilled groups from initial to follow up testing.

	Number of fixations Mean ± SD		Mean duration of fixations Mean ± SD	
Group	Initial	Follow-up	Initial	Follow-up
Skilled	3.35 ± 2.09	4.22 ± 2.14*	1.91 ± 1.23	2.10 ± 1.08*
Less skilled	3.71 ± 2.41	6.10 ± 2.67*	2.04 ± 1.53	2.34 ± 0.99*

*Significantly different to pre-test (*p* < 0.05).

### ROC curve analysis

**Figure 5:**
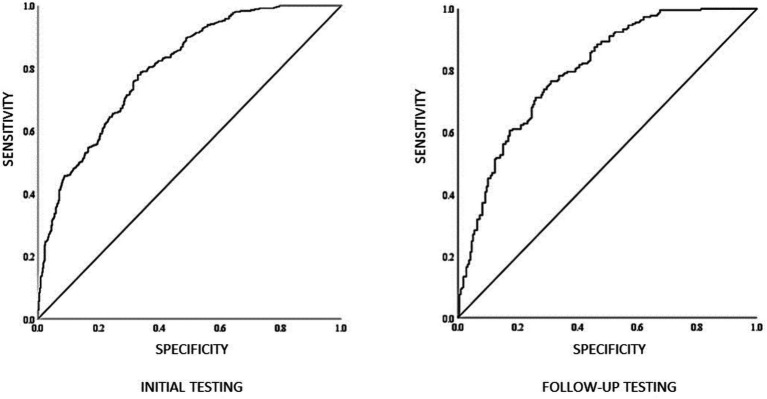
An example of an AUC graph generated from the initial and follow-up data.

An ROC curve was used to test the ability of the combined decision and eye movement measures to classify the skill level of the participants. At the initial testing time point when using the number of fixations, total duration of fixations, and decision time, the area under the ROC curve (AUC) was 0.80, 95%CI [0.770, 0.829]. While at the follow-up when accounting for the number of fixations, total duration of fixation and decision time, the area under the ROC curve was 0.803 95%CI [0.744, 0.862], which highlights an excellent level of discrimination (Figure 5). An AUC between 0.8 and 0.9 is considered excellent discriminability (Mandrekar, 2010).

## Discussion

Traditional TID practices in AR football do not comprehensively assess the tactical, decision-making component required to succeed in elite sporting competitions, instead focusing on the more accessible physical performance measures (Woods et al., 2015). Nor do TID practices consider the influence of maturation stages. Therefore, the purpose of this study was to test a method to examine the decision-making skills and knowledge of junior AR football players using video-based testing and eye movement recording. A within-task skill level classification across two age groups (U14 and U16) was used. A secondary purpose was to examine the stability of decision-making skills and eye movement behavior across an 18-month period by re-testing.

A within-task criterion was used to stratify participants into skilled and less skilled groups based on their decisions and matched against the rankings of a panel of expert coaches. As hypothesized, skilled participants made significantly faster decisions regardless of age (U14/U16, *p* = 0.001). Further, the U16 cohort had significantly faster decision times and were more likely to make skilled decisions compared to the younger cohort (U14). Faster decision times were also evident for the skilled participants during the follow-up testing; however, it should be noted that faster decision-making times were not evident when comparing post (follow-up measurements) to the pre (initial) testing, which may be due to a smaller sample size.

Skilled decision-makers had significantly fewer fixations and shorter total fixation durations, which may be key indicators for the prediction of future skilled decision-making ability or knowledge. Fewer fixations have been associated with more skilled athletes in previous research and shows that skilled athletes are faster and more accurate at identifying and recognizing meaningful patterns in various scenarios while also excluding redundant cues (Laurent et al., 2006; Williams et al., 2011). Moreover, research has highlighted that skilled players are able to recognize and recall structured patterns of play more often (Williams and Ward, 2007), which allows skilled players to differentiate between areas of importance without scanning the whole scene (Mann et al., 2019; Williams and Jackson, 2019).

The shorter total fixation times produced by the skilled group corroborates previous studies in this field which demonstrate that skilled decision-makers develop memory skills that both promote rapid encoding of information in long-term memory and afford selective access to that information when required (Gegenfurtner et al., 2011; Roca et al., 2011, 2014). This means skilled athletes develop more flexible and detailed memory representations than less skilled individuals, allowing them to adapt rapidly to changes in situational demands (Silva et al., 2010).

When measuring performance in junior athletes, the stability of a variable (maintenance of relative rankings over time) is significant for long-term talent prediction. Decision-making accuracy was examined at the follow-up testing session to identify any changes that may have occurred relative to initial testing. Given the additional 18 months of training and practice, it is not surprising that the follow-up group had a higher accuracy score when compared to their previous testing. What is of importance is that regardless of improved response accuracy, the fundamental perceptual-cognitive characteristics remained stable across time. That is, the skilled participants within the follow-up group had a visual search strategy involving fewer fixations and shorter total fixation duration times, similar to the pattern observed in the initial test. The stability of the visual search strategy in the skilled performers over time is unlike physical performance measures, which do not demonstrate stable markers and are less reliable from pre to post-maturation (Cripps et al., 2017). The relatively stable perceptual-cognitive characteristics corroborate previous findings that indicate perceptual-cognitive ability is a by-product of typical training activities such as on-field drills, game simulations, and tactical sessions, e.g., strategic team planning sessions, and not maturation (Williams et al., 2011; Roca et al., 2014).

The ROC analyses also show that the combination of the number of fixations, total duration of fixation, and decision time for the video-based decision-making performance provided an excellent ability to classify players as skilled or unskilled decision-makers. Indeed, Farahani et al. (2020) showed that discrimination between similar skill levels of players was possible when using response time, but not when relying on decision accuracy. Similarly, Dong et al. (2023) show that video-based decision testing may test declarative game knowledge rather than decision-making *per se*. The absence of a perception-action link in the video-based decision-making paradigm is a limitation. It should also be noted, however, that recent literature is inconclusive regarding the superiority of perception-action coupling over perception-only tasks in testing performance capabilities (see Huesmann et al., 2021; Kalén et al., 2021). However, we suggest that testing declarative game knowledge is useful for development athletes, particularly if it can discriminate between skill levels while complementing other tools and not adding to physical testing. As Américo et al. (2017) showed, game knowledge is still developing in junior team-sport athletes.

To examine potential changes in decision-making performance and development over time, we examined descriptive data to compare categorization of players based on decision-making skill at follow-up like that done during the initial testing. This allowed us to compare if players remained in a skilled or less skilled group or changed between testing sessions. Of the 11 players categorized as skilled in initial testing, five performed at a skilled level in the follow-up testing. Of the seven less skilled decision makers who were tested at follow-up, only two remained classified as less skilled, with the remaining five performing better at follow-up. It should be noted, however, that there was a higher accuracy in the follow-up skilled group (78.33%) compared to the percentage for those in the skilled group (70.43%) at initial testing. These comparisons provide some context, however, the data should not be overinterpreted, given the smaller sample at the follow-up testing, thus changes in the comparison pool between initial testing (*n* = 59) and follow-up (*n* = 18). Nevertheless, changes in performance between testing sessions are expected. For example, we expected a level of familiarity with the test at the follow-up test point however, as Dong et al. (2023) point out, even if declarative game knowledge was tested, it is appropriate that this should develop, and facilitate athletes when working with coaches, game plans, and video review sessions. These are worthy assessments for TID and allow a means to identify aspects of players’ potential ability. Additional support for this rationale was found in studies that used a within-group comparison and revealed differences in football players (Vaeyens et al., 2007; Savelsbergh et al., 2010).

While the study was limited to junior male athletes, given the study goals, future research should use longitudinal measures to track both male and female elite junior athletes as they transition into elite senior athletes and measure changes to perceptual-cognitive characteristics. This will enable comparison between sex, where there may be differences in the level of exposure to overall sports training, including decision-making training, and resourcing for academy systems (see MacMahon et al., 2024 for a discussion of the development of expertise in females). A longitudinal approach can also compare drafted vs. non-drafted players and provide greater insight into whether the stability of eye-movement behavior is a key distinguisher. Further, the current study used a film-based task which as acknowledged, may not reflect on-field abilities thus studies that include *in situ* designs will provide deeper knowledge on this topic (Araujo et al., 2006). Lorains et al. (2013) created a custom-designed decision-making notational analysis system to examine the transfer of video-based training to on field competitions. The system considered game context, decision-making (quality, number of options, pressure), and execution (disposal type, effectiveness, error direction) in its performance analysis. In the Lorains et al. (2013) study, the notational analysis was used to compare film-based training to on field performance. Given Lorains et al.’s small sample size, the results are limited, thus future research should look to develop a transfer test in conjunction with film-based objective tools when assessing tactical skill components within AR football. While no significant transfer effects were present in Lorains et al., developing the research in this way is promising.

To conclude, the skills needed to achieve excellence in invasion ball sports are complex and multifaceted, highlighting the importance but also the challenges associated with developing comprehensive and objective TID assessment tools that consider all determinants of gameplay. This study was able to show a viable method for initial screening to identify the key stable visual search behaviors used by junior AR football players for declarative game knowledge and decision-making using the same film-based protocol applied to elite senior players. Further, the visual search characteristics were shown to be a strong, stable marker across time, suggesting that they may be used to objectively measure the tactical skill processes for AR football. This method, in addition to couple skill tests that require perception-action coupling under temporal constraints, can thereby be used to identify players with an elevated level of decision-making and processing skill.

## Data availability statement

The raw data supporting the conclusions of this article will be made available by the authors, without undue reservation.

## Ethics statement

The studies involving humans were approved by the Western Sydney University Human Ethics Committee. The studies were conducted in accordance with the local legislation and institutional requirements. Written informed consent for participation in this study was provided by the participants’ legal guardians/next of kin.

## Author contributions

KS: Writing – review & editing, Writing – original draft, Validation, Supervision, Resources, Methodology, Investigation, Funding acquisition, Formal analysis, Data curation, Conceptualization. LK: Writing – review & editing, Writing – original draft, Validation, Software, Project administration, Methodology, Investigation, Formal analysis, Data curation. SD: Writing – review & editing, Writing – original draft, Validation, Supervision, Methodology, Investigation, Formal analysis, Data curation. BP: Writing – review & editing, Writing – original draft, Supervision, Methodology, Investigation, Formal analysis. JQ: Writing – review & editing, Writing – original draft, Supervision, Resources, Project administration, Methodology, Investigation, Funding acquisition, Formal analysis, Conceptualization. CM: Writing – review & editing, Writing – original draft, Supervision, Resources, Methodology, Investigation, Formal analysis, Data curation, Conceptualization.
